# Levothyroxine-induced serum free thyroxine response following radioactive iodine administration in patients thyroidectomized for differentiated thyroid cancer: A randomized controlled trial

**DOI:** 10.1007/s12020-022-03110-y

**Published:** 2022-06-25

**Authors:** Michela Marina, Giuseppe Maglietta, Giuseppina De Filpo, Rosalia Aloe, Cecilia Gnocchi, Elisa Iezzi, Caterina Caminiti, Graziano Ceresini

**Affiliations:** 1 SSD Medicina interna ad indirizzo onco-endocrinologico, Università di Parma - Azienda Ospedaliero-Universitaria di Parma, Parma, Italy; 2 UO Ricerca clinica ed epidemiologica, Azienda Ospedaliero-Universitaria di Parma, Parma, Italy; 3 SSD Biochimica ad elevata automazione, Azienda Ospedaliero-Universitaria di Parma, Parma, Italy; 4 UO Programmazione e Controllo di Gestione, Azienda Ospedaliero-Universitaria di Parma, Parma, Italy

**Keywords:** Levothyroxine, Free thyroxine, Radioactive iodine, Thyroid cancer

## Abstract

**Purpose:**

Patients undergoing thyroidectomy for differentiated thyroid cancer (DTC) may require 131-radioactive iodine (RAI) administration for remnant ablation or disease treatment. After ingestion, RAI resides within the gastrointestinal tract potentially leading to mucosal damage and abnormalities in the absorption of levothyroxine (LT4). The aim of this study was to evaluate whether serum FT4 peak, induced by a LT4 challenge, changes according to the LT4 formulation (solid or liquid) in both RAI and non-RAI-treated DTC patients.

**Methods:**

This was a monocentric controlled clinical trial, with a parallel two-groups (1:1) randomization of sequence of LT4 formulation. Patients received 200 mcg LT4 orally administered at 08:00 h, in both solid and liquid formulation, at one-week interval, at baseline and after 1, 3, and 6 months from RAI administration. At each time-point, circulating FT4 was evaluated both before LT4 assumption as well as after 1 and 3 h. FT4 increments were evaluated as area under the curve response (AUC). Analogous protocol with the same time-intervals was followed for non-RAI patients.

**Results:**

The trial included 29 consecutive DTC patients, nineteen of whom were submitted to RAI. In RAI subjects, we observed an overall significant reduction in serum FT4 increments with the most relevant decrease at the 1-month time-point, (FT4 AUC: 4.46 ± 0.72 (M ± SD) vs 4.07 ± 0.63 in baseline vs 1-month, *P* = 0.001) without any difference between the two LT4 formulations. No difference in serum FT4 AUC was found in non-RAI subjects.

**Conclusion:**

LT4-induced serum FT4 responses are reduced following RAI administration in thyroidectomized DTC patients.

## Introduction

Thyrotropin (TSH) suppression may represent the main initial goal of levothyroxine (LT4) treatment in patients affected by differentiated thyroid cancer (DTC) who have undergone thyroidectomy followed by radioactive iodine (RAI) administration with the aim to decrease the risk of recurrence [1–4].

The absorption of LT4 takes place in the duodenum, jejunum, and ileum [5, 6]. Several conditions may affect LT4 absorption, such as malabsorption, gastro-intestinal infectious diseases, gastritis, drugs, food [7–12]. The daily dose of LT4 required for optimal treatment may vary in different patients as well as in different conditions [13].

It has been demonstrated that after ingestion, 131Iodine (131I) resides within the gastrointestinal tract [14]. This may lead to a damage of gastrointestinal mucosa and determine, at least theoretically, a modification in the overall phenomenon of absorption.

The type of LT4 formulation has been demonstrated to influence its absorption, with some liquid solutions being more easily absorbed as compared to other formulations [15, 16]. In line with this observation are the reports of better therapeutic results of liquid as compared to solid L-T4 formulation [17–20].

Whether solid and liquid LT4 formulations produce different circulating concentrations of free thyroxine (FT4) after RAI in patients thyroidectomized for DTC is not known.

The aim of this study was to evaluate whether LT4-induced increases in serum concentrations of FT4 are modified according to the LT4 formulation (solid or liquid) in both RAI and non-RAI-treated DTC patients.

## Materials and methods

### Trial design

This was an open-label, monocentric controlled clinical trial, with a randomization of sequence of LT4 formulation (liquid and solid or solid and liquid). The two formulations were administered one week apart from each other, which was considered an appropriate wash-out time.

The protocol included both patients who needed RAI and patients who did not, according to an open design, since it was not possible to blind this sequence of patients due to ethical reasons. The indications for RAI administration were selectively identified following the 2015 ATA guidelines [4].

### Participants

Inclusion criteria were age >18 years, total thyroidectomy with histological diagnosis of DTC, signed informed consent to participate in the study. RAI patients were eligible only if they were deemed to be pre-treated with recombinant human TSH. Exclusion criteria were presence of chronic inflammatory gastrointestinal disease, malabsorption, chronic gastritis, previous external beam radiation, pregnancy, chronic kidney disease, chronic inflammatory or autoimmune disease, treatment with estrogens, selective estrogen receptor- modulators or with drugs known to interfere with LT4 absorption, such as proton pump inhibitors, sucralphate, resins, iron, calcium, alluminum hydroxide, immunosuppressant or chemotherapeutic agents.

### Intervention

With an interval of four to six weeks from thyroidectomy, while on a regular daily treatment with solid formulation of LT4 at a dose estimated to control serum TSH according to ATA guidelines [4], patients were submitted to blood withdrawal after an overnight fast for measurement of circulating FT4 and randomized to receive, instead of the regular daily dose of LT4, a single dose of 200 mcg LT4 either in solid (Tirosint® tablets, IBSA Farmaceutici Italia srl, Lodi, Italy) or liquid (Tirosint® oral solution, IBSA Farmaceutici) formulation. Since data on circulating FT4 after an oral intake of 200 mcg L-T4 are not available in the literature, we based our methods on data obtained with higher L-T4 oral load [15]. According to these studies, the increments in circulating concentration of thyroxine following LT4 oral load occur starting from the first hour from the intake, reaching the maximal difference from baseline at the subsequent 3^rd^ and 4^th^ hour. Therefore, patients had their blood drawn again at the 3^rd^ and 4^th^ hour from LT4 intake for serum FT4 evaluation while the patients were still fasting. The same procedure was repeated after one week, reverting the order of 200 mcg LT4 formulation with respect to the formulation used for the first test (i.e., patients who received solid LT4 at the first testing received liquid LT4 and vice-versa). While continuing their therapeutic daily dose of solid LT4, patients then underwent RAI following human recombinant TSH administration as requested according to guidelines. Patients were then re-tested, according to the above-described procedures 1-, 3-, and 6-months following RAI. The order of the type of LT4 formulation to be administered in these subsequent testing was done based on the sequence resulting from the initial randomization (i.e., patients who started the study with liquid formulation as the first testing maintained this formulation as the first-one in all the testing procedures up to the six-month testing).

Patients who did not receive RAI followed the same procedures, with the time-interval between testing being the same as for patients receiving RAI.

Throughout the study, subjects were on daily treatment with solid LT4. TSH was maintained suppressed in patients who were given RAI and at 0.5–2.0 mIU/L in those who were not, as indicated by guidelines [4].

Circulating FT4 concentrations were measured by chemiluminescence (DIX800 Beckmann instrument) with a functional sensitivity of 0.25 ng/dl and an analytical sensitivity of 0.01 ng/dL, according to the manufacturer’s description of the assay. The intra- and inter-assay coefficients of variation were less than 5% and 10%, respectively.

Blood withdrawal and FT4 assay were done in a double-blind manner with respect to the LT4 testing.

### Ethical aspects

The study was conducted in accordance with the Declaration of Helsinki and the Good Clinical Practice Guidelines of the International Conference on Harmonisation. The protocol was approved by the ethics committee for Parma, Italy (Protocol N. 31871; approval, September 23, 2014) and by the Italian Drug Agency (AIFA) (EUDRACT N: 2014000574-19).

All participants provided prior written informed consent.

### Primary outcome

The aim of this study was to evaluate whether serum FT4 peak, induced by a LT4 challenge, change according to the LT4 formulation (solid or liquid) in both RAI and non-RAI-treated DTC patients.

### Secondary outcomes

The secondary outcomes included the comparison of the LT4 challenge-induced FT4 increments among the different time points of the study (i.e., baseline, 1, 3, and 6 months from RAI or analogous time intervals for non-RAI patients) regardless of the LT4 formulation used for the challenge. Furthermore, the comparison of the two LT4 formulations (i.e., solid and liquid) on the LT4 challenge-induced FT4 changes at all the time-points (i.e., baseline, 1, 3, 6 months) was evaluated.

### Sample Size

Since no reference data were available in the literature, sample size was based on feasibility. Since in the year which preceded the study 26 DTC patients were referred to our Unit, 9 of whom at low risk, we estimated to enroll about 30 subjects in 18 months.

### Randomization and allocation concealment

The choice regarding the formulation of LT4 (i.e, solid or liquid) to be used for each patient as first challenging test was established based on a randomization list and was generated through SAS ver. 8.2 software (SAS, Cary, NC, USA).

The sequence of randomization and the consequent sequence of allocation were centralized and kept blinded until assigned to the patient.

### Statistical methods

To describe patient characteristics, absolute and relative frequencies were used for categorical variables and mean ± standard deviation for continuous variables also including the variation coefficient and geometric mean with the corresponding range for the FT4 serum concentrations. A boxplot was used to graphically depict the distributions of values for FT4 serum concentration levels between RAI and non-RAI subjects from baseline to 1 month. The primary outcome was evaluated by means of the analysis of variance (ANOVA) for repeated measures with interaction term. The changes in FT4 serum concentration levels were expressed as Area Under the Curve (AUC) from baseline to + 3 h (AUC 0–3) which was calculated by using the linear trapezoidal rule method.

Other ANOVA models were implemented to evaluate the secondary outcome, (i.e., the comparison between the two LT4 formulations on FT4 AUC at all study time-points); Tukey test with Bonferroni p-value adjustment was performed to evaluate the interaction term represented by the intersection of the two treatment groups and the four study time points. All statistical analyses were performed by using R-Cran Statistical software, v. 4.03.

## Results

**Fig. 1:**
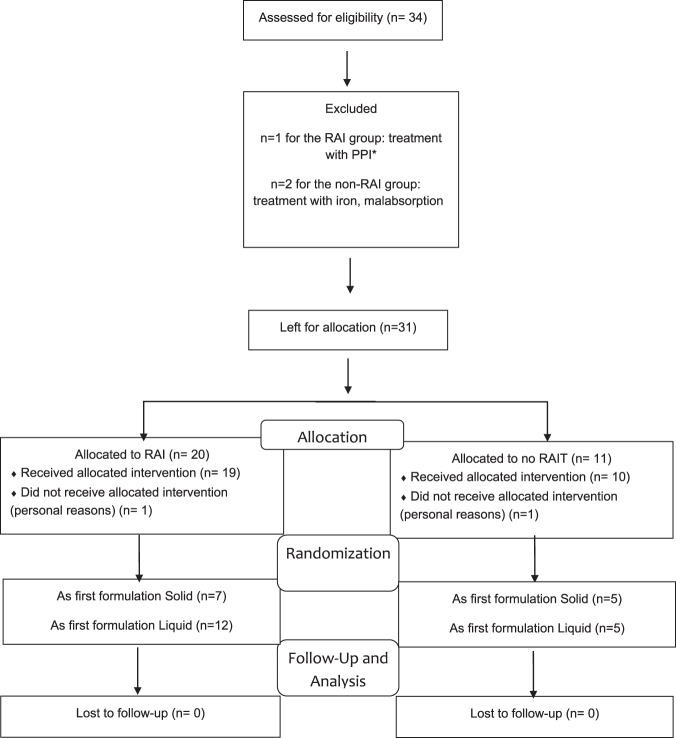
Flow-diagram of enrollment, randomization and follow-up. *PPI Proton pump inhibitors

A flow-diagram of enrollment, randomization and follow-up is reported in Fig. 1. Thirty-one consecutive patients, referred to our Institution after total thyroidectomy for DTC, were enrolled from January 2015 to December 2017. Seventy-six percent of patients were female. Postmenopausal age was documented in 5 and 1 women of the RAI and non-RAI group, respectively. None of the postmenopausal women were on hormone replacement therapy. Twenty patients needed RAI for remnant ablation or treatment of lymph node metastases and eleven did not because of very low-risk disease.

In the RAI group, one patient left the study after the one-month time point because of logistic difficulties. In the non-RAI group, one patient withdrew before the beginning of the study because of personal decision. Therefore, data presented here concern 19 and 10 patients belonging to the RAI and non-RAI group, respectively.

Patient demographic characteristics are reported in Table 1. The majority of the histological types was represented by follicular variant (FV) of papillary thyroid carcinoma (PTC) and PTC in RAI- and non-RAI group, respectively. In 3 cases, lymph node metastases were present at diagnosis in the central compartment. In 11 cases, minor extrathyroidal extension was diagnosed at the histological evaluation. No significant difference in the frequency of DTC histological type was found between RAI and non-RAI patients.

**Table 1:** Patients’ characteristics

	RAI (19)	NO RAI (10)	*p*-value
Age mean ± SD	47.26 ± 11.43	51.70 ± 13.35	0.01
Sex F, *N* (%)	17 (89.5%)	5 (50%)	<0.001
Histological type			0.083
PTC N (%)	3 (15.8%)	7 (70%)	
FVPTC N (%)	12 (63.1%)	2 (20%)	
PTC other variants N (%)	4 (21.1%)	0	
FTC N (%)	0	1 (10%)	
FT4 AUC 0–3 baseline			0.01
mean ± SD (CV%)	3,69 ± 0,51 (13,9%)	4,46 ± 0,72 (16,4%)	
geometric mean (Range)	3,65 (2,74–4,75)	4,40 (3,27–5,94)	

All patients were operated on by the same surgical team. In the RAI group, ultrasound detectable (US), scan positive, remnant thyroid tissue was found in 2 patients with a maximum diameter of 5 mm; thirteen patients had no US remnant tissue but they had a post-dose, faint, scan positivity in the thyroid bed; in 4 patients, neither US nor scan positivity were found. In the non-RAI group, 1 patient had a US remnant tissue of 4 mm.

The mean dose of 131I administered to the RAI patients was 2590.0 ± 1226.92 (M ± SD) MBq.

No patient had severe clinical consequences of RAI; in 10 patients, a modest bilateral parotid discomfort was observed which required no treatment.

Four and 2 patients in the RAI and non-RAI group, respectively, were hypothyroid before surgery.

Patients regularly took their therapeutic daily dose of l-thyroxine in a fasting condition with 1 h interval between LT4 assumption and the subsequent breakfast.

The day in which patients were tested with 200 mcg of LT4, fasting condition was maintained up to the last blood sample (i.e., up to the third hour from 200 mcg LT4 ingestion, only water being allowed). The mean therapeutic daily dose of LT4 was 2.02 ± 0.21 (M ± SD) and 1.93 ± 0.11 mcg/Kg/bw in RAI and non-RAI group, respectively (*P* = 0.062).

**Fig. 2:**
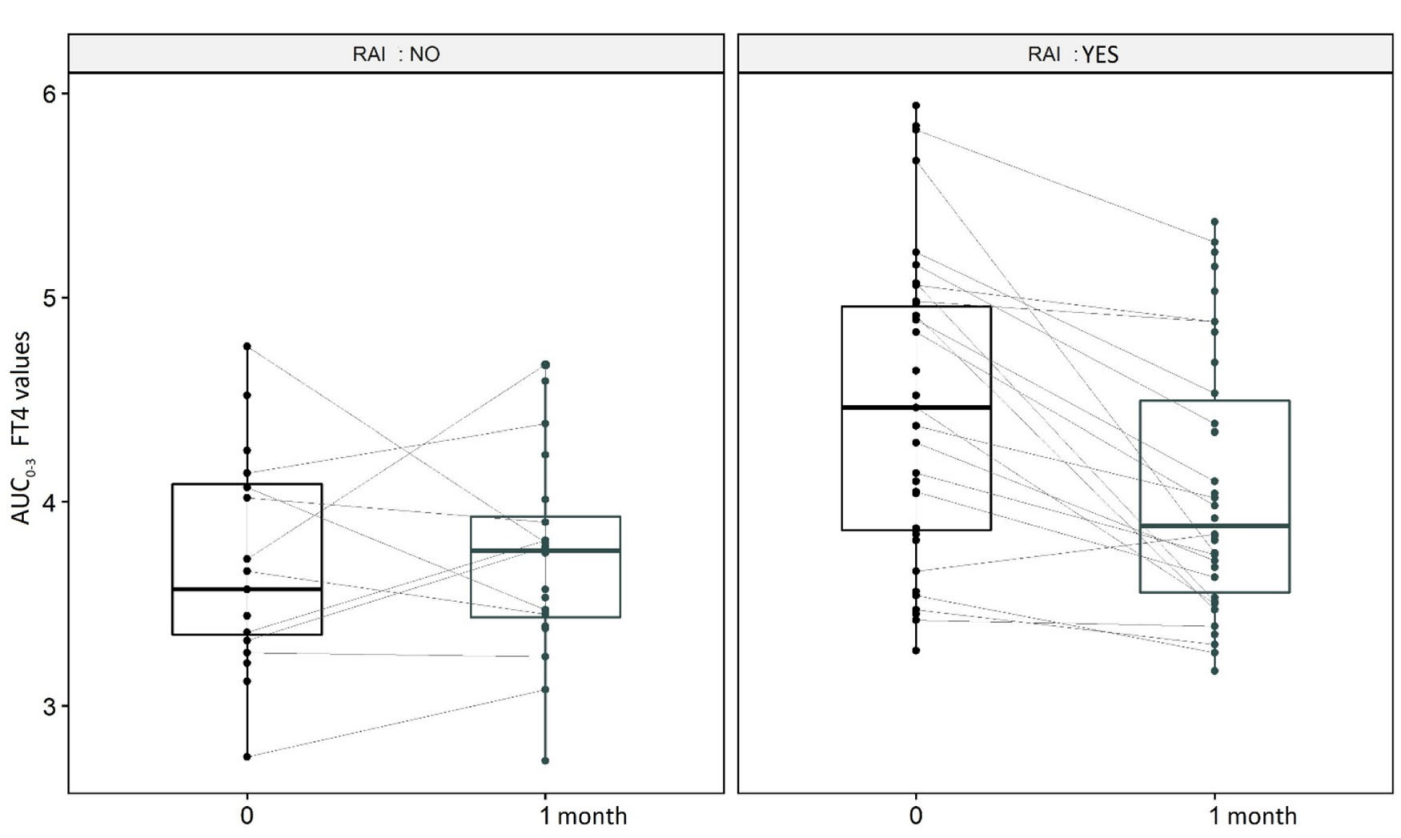
Boxplot of *AUC*_0–3_ FT4 values at Baseline and 1 month among RAI and NO RAI groups. The box is delimited by the first and the third quartile in which, the bold horizontal line represents the median value. The slight conjunction lines between couple of points on the boxplot represents the change in FT4 values of a specific patient from baseline to 1 month

Regarding the primary outcome, the analysis of the LT4 challenge induced FT4 responses from baseline to 1 month in the 2 groups (i.e., RAI and non-RAI), regardless of the LT4 formulation, showed a significant t difference both between groups (*p* = 0.014), and through time (*p* < 0.001). In particular, the interaction term (*β*_Groups x Time_ = −0.44, *p* = 0.004) indicates a significant decrease of FT4 concentration values at 1 month in the RAI group (Table 2). Conversely, in the non-RAI group a slight, nonsignificant, mean increase was observed, as also shown in Fig. 2.

**Table 2:** ANOVA for repeated measures (Baseline, +1 month) with interaction term (Group x Time) model

	Df	Sum Sq	Mean Sq	*F*-value	Pr(>F)
Error: ID					
RAI	1	8.28	8.278	6.992	0.0135*
Residuals	27	31.96	1.184		
Error: Within					
TIME	1	1.707	1.7066	12.044	0.000819***
RAIxTIME	1	1.276	1.2756	9.002	0.003538**
Coefficients					
	No-RAI	RAI			
	3.703250	4.265263			
Within:	TIME:1month			RAIx1month	
	0.0465000			−0.4412368

**Fig. 3:**
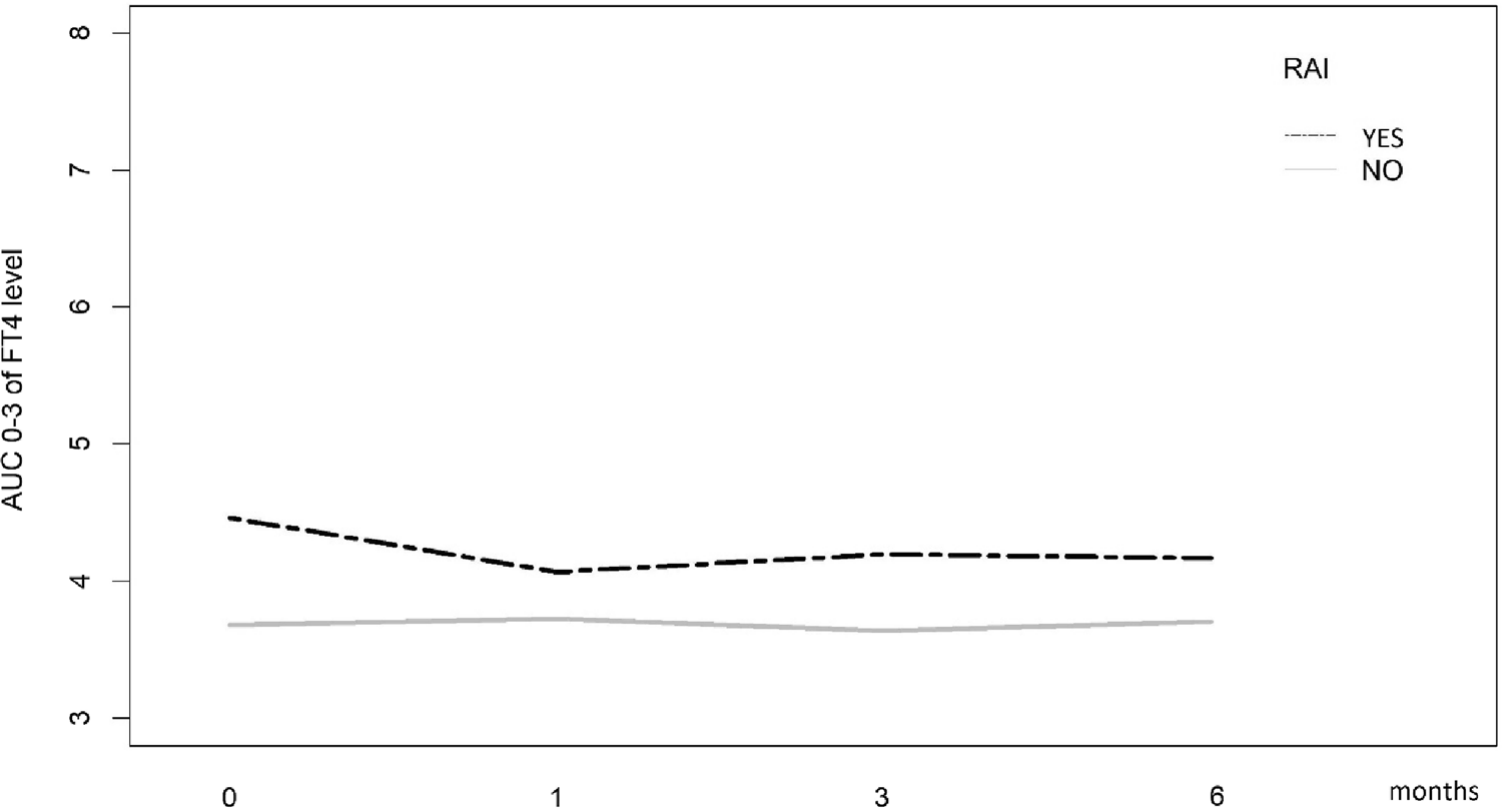
Trajectories of *AUC*_0–3_ FT4 values determined by the linear conjunction of mean values calculated at Baseline, 1, 3, and 6 months into the 2 groups (RAI and NO RAI)

Concerning the secondary outcome, when changes in the LT4 challenge-induced FT4 increments were evaluated in both RAI and non-RAI group at the different study time-points, regardless of the LT4 formulation, the results of the ANOVA model (Table 3) confirmed the significance for each factor (group, time-point) and for the interaction (*p* = 0.046). These results are also shown in Fig. 3 in which FT4 concentration values appeared stable in the non-RAI group between the times of administration, whereas in the RAI group a significant decrease was observed, except for the value at +3 months. These trends were analyzed through multiple comparisons of means, performed by Tukey test (Supplementary Fig. 1), in which the most relevant decrease in FT4 concentration values was confirmed at 1 month from baseline in the RAI group (*p* < 0.001).

**Table 3:** ANOVA for repeated measures (Baseline, +1, +3, +6 months) with interaction term (Group x Time) model

	Df	Sum Sq	Mean Sq	*F*-value	Pr(>F)	
Error: ID						
RAI	1	15.06	15.06	8.099	0.00835**	
Residuals	27	50.21	1.86			
Error: Within						
TIME	3	1.97	0.6552	3.894	0.00986**	
RAIxTIME	3	1.37	0.4567	2.714	0.04605*	
Coefficients						
	No-RAI	RAI				
	3.686875	4.222961				
Within:	TIME:1month	TIME:3month	TIME:6month	RAIx1month	RAIx3month	RAIx6month
	0.0465000	−0.0432500	0.0242500	−0.4412368	−0.2249079	−0.3200395

**Fig. 4:**
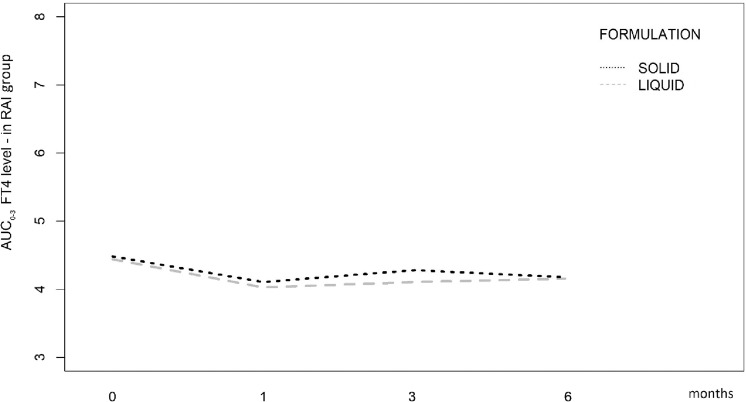
Trajectories of *AUC*_0–3_ FT4 values determined by the linear conjunction of mean values calculated at Baseline, 1, 3, and 6 months into the 2 formulations of LT4 (SOLID and LIQUID) into the RAI group

When the curves of FT4 AUC were compared between solid and liquid formulations within the RAI group, no statistically significant difference was observed (Fig. 4).

## Discussion

The results of this study demonstrate that the increments in FT4 circulating concentrations following a 200 mcg-LT4 challenge are significantly reduced after RAI administration. We observed that this phenomenon is still present at the sixth month of observation, although the most significant results are found at the first month following RAI.

Several reports suggest that RAI results in a disruption of the integrity of the mucosa of the gastrointestinal tract. This may lead to the alteration of the absorption of several substances. Whether this also applies to LT4 is not known.

We found that the decrease in circulating FT4 observed after RAI was not related to the formulation of LT4 we used for the challenge. Liquid LT4 formulation represents an important alternative to the use of solid formulations in LT4 treatment. This is highlighted by the increased bioavailability of liquid, versus solid, LT4 formulation, which has been well-demonstrated in physiological as well as pathological conditions [21]. However, little is known about the use of liquid versus solid LT4 formulations in patients undergoing RAI following total thyroidectomy for DTC. In 2014, Giusti et al. reported no change in TSH, thyroid hormones or thyroglobulin during either liquid or tablet formulations of LT4 in a 70-day survey of DTC patients although liquid LT4 formulation produced a better stability of TSH levels as compared to tablet formulation [22]. However, that study included patients who had undergone RAI remnant ablation and others who had not. Moreover, RAI was administered about 10 years earlier with respect of the date of the study, and, finally, the length of the observation was quite short. In 2017, Cappelli et al. demonstrated that among patients who were thyroidectomized and RAI-ablated for DTC, those who were on liquid LT4 therapy obtained a better control of TSH as compared to those on solid LT4 formulation [23]. However, in that study the adherence to the protocol was assessed through personal interviews with intervals of 8 to 12 months and no data were provided regarding serum TSH right after RAI. In our study we evaluated the rise of circulating FT4 following either liquid or solid LT4 challenge in DTC patients at different time intervals after RAI administration in order to evaluate possible effects of the time elapsed from RAI administration and the type of formulations of LT4 and to provide a more detailed information on post-RAI circulating LT4 peaks.

Based on our results, one could expect that the standard daily therapy of our patients during the whole study, failed to completely suppress TSH during the six-month follow-up in RAI patients. This point is difficult to be addressed because, as often observed in these patients, the TSH-suppressive LT4 dose prescribed soon after thyroidectomy and RAI administration is frequently empirically determined and may be higher than that required. Indeed, the first weeks or months of such treatment represent a period of a titration dose of LT4 to be administered. For this reason, our patients are seen every two to three months after RAI to adjust LT4 dose, based on TSH levels. However, it might be interesting to note that at the six-month follow-up, there was a tendency toward an increase in serum TSH in 8 RAI patients with values being over the suppression cut-off in 4 of them. Conversely, non-RAI subjects, exhibited good TSH control with no subjects displaying circulating TSH over the cut-off values for the duration of the study, the values being even close to the suppression cut-off, for some of them. We did not find any relationship between baseline serum TSH and the results of the LT4 challenge test (data not shown), although the above-mentioned observations on the serum TSH trajectories in both groups, as well as the low number of patients, may have interfered with these data.

We found that at baseline, the increase in FT4 after LT4 challenge was lower in non-RAI patients as compared to RAI patients. This may be due to the different characteristics between RAI and non-RAI patients according to their different clinical risk profiles.

Another possible explanation could reside in the fact that non-RAI patients had a tendency toward an increase in body mass index (23.71 ± 2.16 vs 25.32 ± 1.99 in RAI vs non-RAI patients, *P* = 0.77, data not shown) so that, higher amount of LT4 could be needed to reach the peak observed in RAI patients. Also, the lower dose of LT4 in non-RAI patients and the small sample size, unbalanced between RAI- and non-RAI patients, might have contributed to this phenomenon.

However, the perspective is that the similar FT4 AUC observed in both groups starting at the one-month evaluation is mainly due to the decreased LT4-induced FT4 response in RAI patients, with the curve displaying FT4 AUC at each timepoint remaining flat throughout the study in non-RAI patients.

It is well-known that the dose of LT4 needed to suppress TSH may vary from patient to patient and doses up to 2.6 mcg/kg bw/day have been reported with even further increments [24, 25]. For this reason, we considered the 200 mcg dose as a useful challenge to test the increase in circulating FT4 after LT4 intake in our patients because this dose may represent (or may be close to) a therapeutic dose at least in some patients.

Studies on LT4 absorption have been reported in the literature with an oral load of LT4 of 600–1000 mcg [26]. In our study, we tested the increase in FT4 after the administration of a lower dose of LT4. However, our study cannot be considered a specific study on the absorption of LT4, even though we believe that the ability of the FT4 assay we used in our study to discriminate between 0.01 mcg of FT4 is sufficient to make our data clinically reliable. In this context, to our knowledge, this is the first study on LT4-stimulated FT4 peak concentrations after RAI administration.

A few women of both RAI and non-RAI groups were in postmenopausal age and none of them were on hormone replacement therapy. Therefore, we believe that an effect of menopausal status on our results is unlikely to have occurred.

A few patients of both groups had small US thyroid tissue remnants. We have no data to define to what extent these small amounts of thyroid tissue had a significant impact on the basal circulating concentrations of FT4. However, we believe that the low entity of this phenomenon is unlikely to have affected our results.

One strength of this study was the randomization of sequence of administered LT4 formulations, which improved study efficiency and its feasibility. Moreover, centralization of analyses in a single laboratory provided a standardized assessment method and performance of all assays at the same laboratory. This study has some limitations. First, it included a relatively small number of patients enrolled in only one center, which may limit result generalizability. Second, the washout period between administrations of the two formulations of LT4 may have been too short. However, we chose the 1-week interval between the two LT4 challenge based on the half-life of LT4 which has been reported to be approximately 7 days [27]. This likely kept basal circulating LT4 interference low at each test. Also, it has to be considered that longer intervals between challenges would have caused a prolongation of the study. This would not have been ethically acceptable for patients who were waiting for radioactive administration. We also want to acknowledge that long time elapsed from the end of enrollment to manuscript submission; this was due to organizational difficulties, including the recent pandemic health emergency.

In conclusion, our results suggest that in thyroidectomized DTC patients the FT4 increase following LT4 administration may be reduced in the first months after RAI, regardless of the LT4 formulation.

These data may be useful to advance knowledge on LT4 therapeutic management of DTC patients.

## Supplementary Information

Supplementary Information
