# Supplementary material for: Levothyroxine-induced serum free thyroxine response following radioactive iodine administration in patients thyroidectomized for differentiated thyroid cancer: A randomized controlled trial
Source: Endocrine. 2022 Jun 25;77(2):340–8. doi: 10.1007/s12020-022-03110-y (PMC9325824; doi:10.1007/s12020-022-03110-y)

Supplementary Figure 1

Multiple Comparisons of means by Tukey Contrasts of ANOVA for repeated measures (Baseline, +1, +3, +6 months) with interaction term (Group x Time) model. P values reported are Adjusted by Bonferroni method.


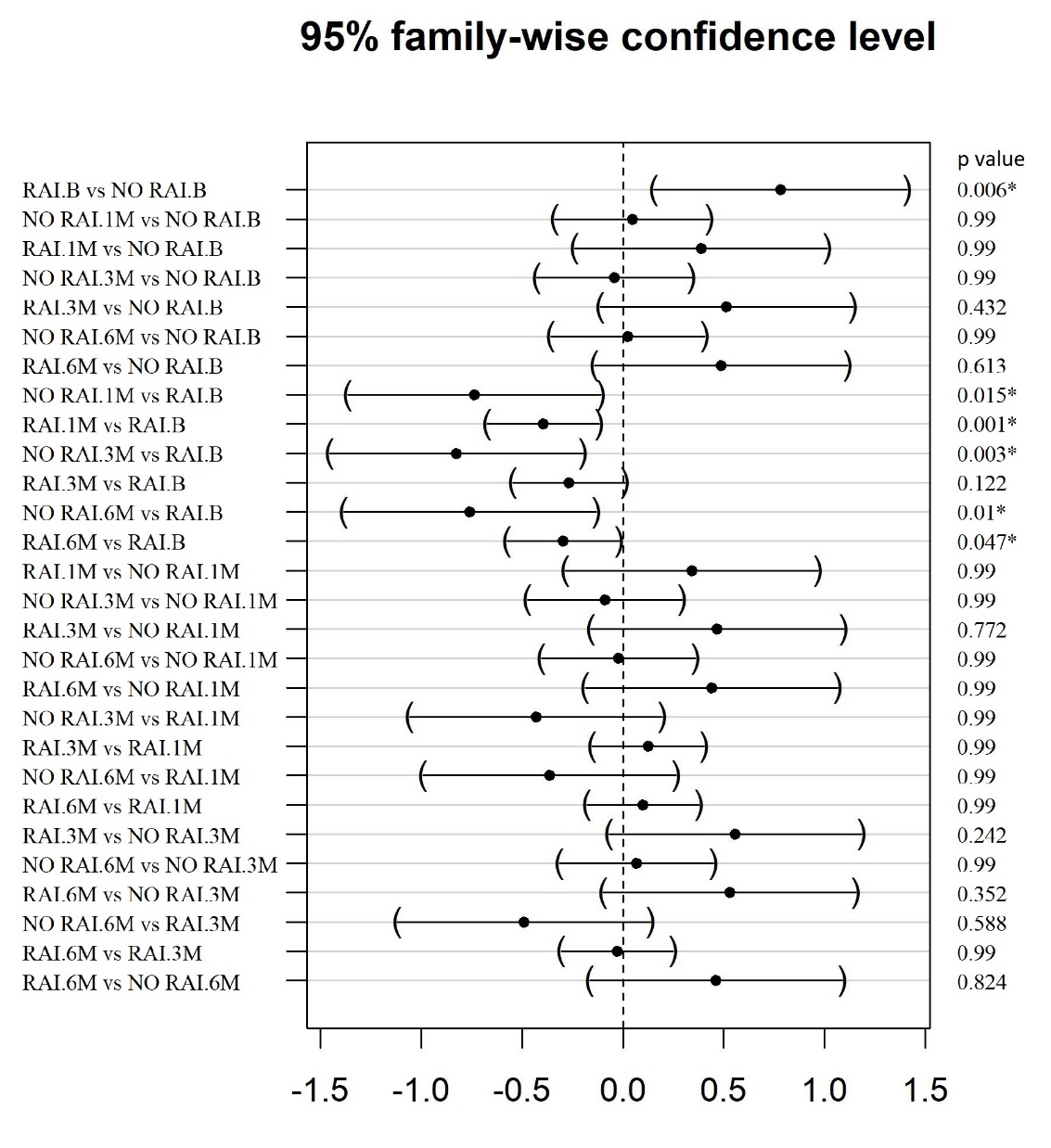

Supplement: Supplementary file 1 — Supplementary Information [file 12020_2022_3110_MOESM1_ESM.docx]
